# Rapid cycling bipolar disorder is associated with antithyroid antibodies, instead of thyroid dysfunction

**DOI:** 10.1186/s12888-019-2354-6

**Published:** 2019-12-02

**Authors:** Zhaoyu Gan, Xiuhua Wu, Zhongcheng Chen, Yingtao Liao, Yingdong Wu, Zimeng He, Zhihua Yang, Qi Zhang

**Affiliations:** 1 Department of Psychiatry, the Third Affiliated Hospital of Sun Yat-sen University, Guangzhou, China; 2 Clinical laboratory, the Third Affiliated Hospital of Sun Yat-sen University, Guangzhou, China; 3 Biotherapy Center, the Third Affiliated Hospital of Sun Yat-sen University, NO.600, Tianhe Road, Tianhe District, Guangzhou, 510630 NO China

**Keywords:** Bipolar disorder, Rapid cycling, Thyroid dysfunction, Antithyroid peroxidase antibodies, Anti-thyroiglobulin antibodies

## Abstract

**Background:**

Conclusions regarding the association between antithyroid antibodies or thyroid dysfunction and rapid cycling bipolar disorder (RCBD) have been conflicting. Previous studies suggest that the impact of antithyroid antibodies on mental wellbeing seems to be independent of thyroid function. Here, we investigated their independent association with RCBD in a large, well-defined population of bipolar disorder (BD).

**Methods:**

Fast serum levels of free thyroxine (FT4), free triiodothyronine (FT3), thyroid Stimulating Hormone (TSH), TPO-abs and Tg-abs were simultaneously measured in 352 patients with BD. Clinical features of BD were collected through semi-structural interview conducted by trained interviewers with background of psychiatric education.

**Results:**

Neither hypothyroidism nor hyperthyroidism was significantly associated with RCBD. Both TPO-abs and Tg-abs were significantly related to RCBD, even after controlling for gender, age, marriage status, education, antidepressants treatment, comorbidity of thyroid diseases, and thyroid function (serum levels of FT3, FT4 and TSH). Although TPO-abs and Tg-abs were highly correlated with each other, binary logistic regression with forward LR selected TPO-abs, instead of Tg-abs, to be associated with RCBD. TPO-abs was significantly, independently of Tg-abs, associated with hyperthyroidism, while Tg-abs was marginally significantly related to hypothyroidism at the presence of TPO-abs.

**Conclusion:**

TPO-abs might be treated as a biomarker of RCBD. Further exploring the underlying mechanism might help understand the nature of RCBD and find out new treatment target for it.

## Background

Both thyroid dysfunction and antithyroid antibodies have been widely reported to be associated with affective disorders. Cross-sectional [1, 2] or cohort studies [3, 4] have found that hypothyroidism increased risk of developing depression or bipolar disorder. Thyroid hormones, as an adjunctive treatment, have been proved to be effective in improving the response to ongoing treatment among euthyroid patients with refractory depression [5, 6] or bipolar depression [7]. Further studies indicate that thyroid hormones seem to show modulating effect on the brain serotonin system [8, 9], which might partly explain the role of thyroid hormone in the pathophysiology of affective disorders. At the same time, several studies have found that the prevalence of antithyroid antibodies is higher among patients with depression or bipolar disorder (BD) than general population [10, 11] or those with schizophrenia [12]. Compared to health control, the prevalence of antithyroid peroxidase antibodies (TPO-Abs) in bipolar offspring [13] or co-twins of bipolar cases [14] is also found to be higher, implying BD might share common genetic predisposition with autoimmune thyroiditis.

Among the relationship between autoimmune thyroiditis and bipolar disorder, the association between autoimmune thyroiditis and rapid cycling bipolar disorder (RCBD) is especially paid attention to. A large number of studies show that RCBD is associated with hypothyroidism [15–19]. Furthermore, high dose of levothyroxine is also reported to help stabilize mood among RCBD [20, 21], suggesting hypothyroidism might play a role in the development of RCBD. In addition, a clinical research finds that TPO-abs are related to rapid cycling bipolar disorder [22].

However, not all studies can document such association [10, 23–25]. Moreover, although autoimmune thyroiditis is one of the major cause of thyroid dysfunction, the impact of antithyroid antibodies on mental wellbeing seems to be independent of thyroid dysfunction [26]. Finally, although both TPO-abs and Tg-abs are biomarkers of autoimmune thyroiditis, it is still unclear which dominates the association between autoimmune thyroiditis and BD. Most of previous studies seem to favor TPO-abs [27]. However, a recent study finds the prevalence of Tg-abs (but not TPO-abs) is related to a lower risk of readmission in BD, suggesting Tg-abs might play a part in the association between autoimmune thyroiditis and BD. That is to say, what role TPO-abs, Tg-abs, and thyroid dysfunction play in the association between autoimmune thyroiditis and BD, particular RCBD remains unknown. We hypothesized that TPO-abs, Tg-abs, and thyroid dysfunction might correlate with each other but impose different impact on BD. Thus, in this study, we measured both thyroid function and serum level of antithyroid antibodies among a large, well-defined population of BD, aiming to explore the independent impact of thyroid dysfunction and antithyroid antibodies on RCBD.

## Methods

### Subjects

Cases were drawn from patients who were admitted or had ever admitted for mood disorders to the inpatient service of the Third Affiliated Hospital of Sun Yat-sen University between July 1, 2012 and February 1, 2018. Potential participants were recommended by their treating psychiatrists. Eligible participants met the DSM-IV-TR criteria for BD by using Chinese version of the Structured Clinical Interview for the Diagnostic and Statistical Manual of Mental Disorders, Fourth Edition, Text Revision (DSM-IV-TR) Axis 1 Disorders (SCID-I) [28]. Patients with evidence of organic mental disorder were excluded. Patients who were pregnant or confined in the past 6 months were also excluded. All the subjects were Han Chinese, aged 18 or above, had no currently active severe physical diseases confirmed by routine clinical examination and provided written informed consent. This study were reviewed and approved by the Clinical Research Ethics Committee of the Third Affiliated Hospital of Sun Yat-Sen University.

### Measurement

#### Biochemical measurement

The fasting blood samples were collected between 7:00 AM and 9:00 AM. For inpatient cases, blood drawing was arranged on the next day after admission. All the blood samples were sent to the immunology laboratory of the Third Affiliated Hospital of Sun Yat-sun University to have thyroid function and antithyroid antibodies measured within 2 h after the blood was drawn.

The assays of free thyroxine (FT4), thyroid stimulating hormone (TSH), TPO-abs and anti-thyroglobulin antibodies (Tg-abs) were done by the chemilumniscence (CLIA) method using ADVIA Centaur system (Siemens, Massachusetts, USA). Reagents were also obtained from the Siemens Company. The reference ranges for these assays were listed as the following: FT4 = 11.5–22.7 pmol/l, FT3 = 3.5–6.5 pmol/l, TSH = 0.55–4.78μIU/ml, TPO-Abs or Tg-Abs =0–60 U/ml.

#### Definition of hypothyroidism, hyperthyroidism, TPO-abs positivity and Tg-abs positivity

According to previous reports [29–31], hypothyroidism here was defined as FT4 < 11.5 pmol/l or TSH > 4.78μIU/ml or currently under levothyroxine treatment, hyperthyroidism was defined as FT4 > 22.7 pmol/l or TSH < 0.55μIU/ml. If both FT4 and TSH fell in their reference ranges, thyroid function was considered as normal. TPO-Abs or Tg-Abs was considered as positivity when their titres were above 60 U/ml.

#### Clinical assessment

General sociodemographic and clinical characteristics were collected via a questionnaire designed by the researchers. Diagnostic assessment was performed using the Chinese version of the Structured Clinical Interview for the Diagnostic and Statistical Manual of Mental Disorders, Fourth Edition, Text Revision (DSM-IV-TR) Axis 1 Disorders (SCID-I) [28]. Rapid cycling was determined by examining the number of prior affective episodes in the past 12 months according to the above-mentioned diagnostic assessment. An affective episode was defined as a manic, hypomanic, mixed or depressive mood separated by 8 weeks of euthymia or an episode of an opposite polarity [32]. “Truncated episodes”, which met DSM-IV-TR severity criteria but not duration criteria for an episode of mania, hypomania, or major depression [33], were also counted. Rapid cycling was established once the number of prior affective episodes (including truncated episodes) in the past 12 months reached 4 or above. If truncated episode was included, the patient should be fully symptomatic for at least 8 weeks during the reference year, aiming to exclude those who had been ill for only a few days [33]. Severity of the current episode were evaluated with the 17-item Hamilton Depression Scale (HAMD-17) [34] and the Young Mania Rating Scale (YMRS) [35]. Atypical features were assessed by the corresponding section of the Chinese version of SCID-I based on the patients’ most severe depressive episode they had ever experienced. Comorbidity of thyroid diseases was confirmed by reviewing the patients’ previous medical history and medical records stored in our hospital’s electronic medical system. The above-mentioned interview was conducted by trained psychiatrists in our study team.

### Statistic analysis

The prevalence of hypothyroidism, hyperthyroidism, TPO-abs positivity and Tg-abs positivity were calculated. Subjects were divided into two groups according to whether they had the feature of rapid cycling: rapid cycling (RC) and non-rapid cycling (NRC). For continuous data with normal distribution, independent- samples t-test was used to test the difference between RC and NRC groups. Differences in categorical parameters between groups were tested using Chi-square test. Univariate binary logistic regression was performed to examine the association between hypothyroidism, hyperthyroidism, TPO-abs or Tg-abs and RCBD. Multivariate logistic regression model was used to adjust for potential confounding factors. Spearman’s correlation analysis was administered to test the relationship between TPO-abs and Tg-abs. Odds ratios and 95% confidence intervals were used to quantify the strength of associations. The results were considered significant at *P* < 0.05. All data were analyzed using commercial statistical package SPSS 19.0 (SPSS, Inc., Chicago, IL).

## Results

### The demographic and clinical characteristics of the subjects

Totally, 375 candidates were screened and finally 352 (93.9%) subjects were eligible to be included in this study. Among the 23 ineligible candidates, 9 refused to participate in this study, 10 were suffering from other acting severe physical diseases, 2 were pregnant, and 2 were confined in the past 6 months. The demographic and clinical characteristics of the 352 eligible participants were detailed in the Table 1. Except YMRS total scores were higher in NRC than in RC (*p* = 0.010), no significant difference was found between NRC and RC in age, gender proportion, education, marriage status, psychotic features, atypical features, comorbidity of thyroid diseases, substance abuse, HAMD-17 total scores, BMI, style of current episode, duration of illness and psychopharmaceutical treatment (*p* > 0.05).

**Table 1:** The demographic and clinical characteristics of the subjects

variables	RC^a^ *N* = 46	NRC *N* = 306	Total *N* = 352
Age (mean ± SD) (year)	28.2 ± 14.2	27.8 ± 10.6	27.5 ± 11.6
Female [*n* (%)]	31 (67.4%)	175 (57.2%)	206 (58.5%)
Years of education (mean ± SD) (year)	12.0 ± 3.5	12.5 ± 3.3	12.4 ± 3.3
Marriage status			
Married [*n* (%)]	14 (30.4%)	95 (31.0%)	109 (31.0%)
Single [*n* (%)]	29 (63.0%)	199 (65.0%)	228 (64.8%)
Divorced [*n* (%)]	3 (6.5%)	11 (3.6%)	14 (4.0%)
Widowed [*n* (%)]	0 (0.0%)	1 (0.3%)	1 (0.3%)
Psychotic features [*n* (%)]	9 (19.6%)	66 (21.6%)	75 (21.3%)
Atypical features	*N* = 44	*N* = 297	*N* = 341
Increased appetite [*n* (%)]	6 (13.0%)	68 (22.9%)	74 (21.7%)
Weight gain [*n* (%)]	4 (8.7%)	55 (18.5%)	59 (17.3%)
Hypersomnia [*n* (%)]	14 (30.4%)	81 (27.3%)	95 (27.0%)
Comorbidity of thyroid diseases^a^	5 (10.9%)	14 (4.6%)	19 (5.4%)^b^
Substance abuse [*n* (%)]	2 (4.3%)	22 (7.2%)	24 (6.8%)
HAMD-17 total scores (mean ± SD)	19.4 ± 7.6	18.7 ± 9.5	18.7 ± 9.2
YMRS total scores (mean ± SD)	8.3 ± 8.0	11.8 ± 10.5^*^	11.3 ± 10.4
BMI (mean ± SD)	21.4 ± 3.5	21.8 ± 3.6	21.7 ± 3.5
Current episode			
remission	3 (6.5%)	28 (9.2%)	31 (8.8%)
depressive	27 (58.7%)	144 (47.1%)	171 (48.6%)
(hypo)manic	1 (2.2%)	29 (9.5%)	30 (8.5%)
mixed	15 (32.6%)	105 (34.3%)	120 (34.1%)
Duration of illness (mean ± SD) (year)	5.1 ± 5.7	4.9 ± 6.0	4.8 ± 5.9
Psychopharmaceutical treatment [*n* (%)]	15 (32.6%)	105 (34.3%)	120 (34.1%)
Lithium [*n* (%)]	2 (4.3%)	19 (6.2%)	21 (6.0%)
Anticonvulsants [*n* (%)]	8 (17.4%)	46 (15.1%)	54 (15.3%)^b^
Antipsychotics [*n* (%)]	10 (21.7%)	80 (26.1%)	90 (25.5%) ^c^
Antidepressants [*n* (%)]	9 (19.6%)	38 (12.4%)	47 (13.4%)^d^

*p < 0.05; RC: rapid cycling; NRC: non-rapid cycling; HAMD-17: 17-item Hamilton Depression Scale; YMRS: Young Mania Rating Scale

^a^Including 9 with history of Grave’s diseases, 3 with thyroiditis, 2 with thyroid cyst, 2 with thyroid nodule, 1 with history of thyroid cancer., 3 with primary hypothyroidism.

^b^Including 43 under sodium valproate, 11 under lamotrigine, 8 under oxcarbazepine, 1 under topiramate

^c^Including 54 under Quetiapine, 21 under olanzapine, 7 under aripiprazole, 5 under parpiperazone, 3 under risperdone, 2 under clozapine, 2 under amisulpride, 2 under haloperidol, 1 under ziprasidone,

^d^Including 12 under escitalopram, 9 under venlafaxine, 7 under paroxetine, 3under duloxetine, 5 under fluoxetine,. 4 under fluvoxamine, 3 under mitrazapine, 5 under sertraline, 6 under escitalopram, 2 under bupropion,

### The association between thyroid dysfunction and RCBD

#### Comparison of fast serum levels of FT3, FT4, and TSH between RC and NRC

We first compared the fast serum levels of FT3, FT4, and TSH between RC and NRC in all the participants. As Table 2 demonstrated, RC did not differ significantly from NRC in the serum levels of FT3, FT4 and TSH. However, the variances of FT3, FT4 and TSH in RC were significantly larger than those in NRC (*p* = 0.03, < 0.001, < 0.001 respectively). Then, we did the same comparison after excluding patients with comorbidity of thyroid diseases. It came out that only the variance of FT4 was significantly different between RC and NRC (*p* = 0.002). However, if the above-mentioned comparison was done in female and male participants respectively, the difference in the variance of FT4 between RC and NRC only existed in female participants (*p* < 0.001). Finally, the same comparison was performed again after excluding patients with comorbidity of thyroid diseases or under psychopharmaceutical treatment within 3 months prior to recruitment. The results showed that both the mean and the variance of FT3, FT4 and TSH were not significantly different between RC and NRC (*p* > 0.05).

**Table 2:** Comparison of fast serum levels of FT3, FT4, and TSH between RC and NRC

	FT3 (mean ± SD) (pmol/l)	FT4 (mean ± SD) (pmol/l)	TSH (mean ± SD) (μIU/ml)	*P*^a^	*P*^b^	*P*^c^
^d^RCBD (*n* = 47)	4.9 ± 1.7	19.0 ± 15.4	3.2 ± 7.5	0.982^g^	0.313^g^	0.251^g^
Non-RCBD (*n* = 306)	4.9 ± 0.9	16.7 ± 4.7	1.9 ± 1.6	0.030^h^	< 0.001^h^	< 0.001^h^
^e^RCBD (*n* = 41)	4.7 ± 0.9	19.3 ± 16.0	2.1 ± 1.4	0.982^g^	0.313^g^	0.251^g^
Non-RCBD(*n* = 292)	4.9 ± 0.9	16.6 ± 4.7	1.9 ± 1.5	0.56^h^	< 0.002^h^	< 0.792^h^
^f^RCBD (*n* = 14)	5.0 ± 0.7	17.0 ± 3.0	1.7 ± 1.0	0.842^g^	0.702^g^	0.705^g^
Non-RCBD (*n* = 123)	5.1 ± 1.0	17.6 ± 5.7	1.9 ± 1.9	0.894^h^	0.592^h^	0.485^h^

*RCBD* Rapid cycling bipolar disorder

^a^For FT3

^b^For FT4

^c^For TSH

^d^Including all the patients

^e^Excluding patients with comorbidity of thyroid diseases

^f^Excluding patients under pyschopharmaceutical treatment within 3 months prior to recruitment or with comorbidity of thyroid diseases

^g^Test for equality of means

^h^Test for equality of variance

#### The prevalence of hypothyroidism and hyperthyroidism in BD

**Fig. 1:**
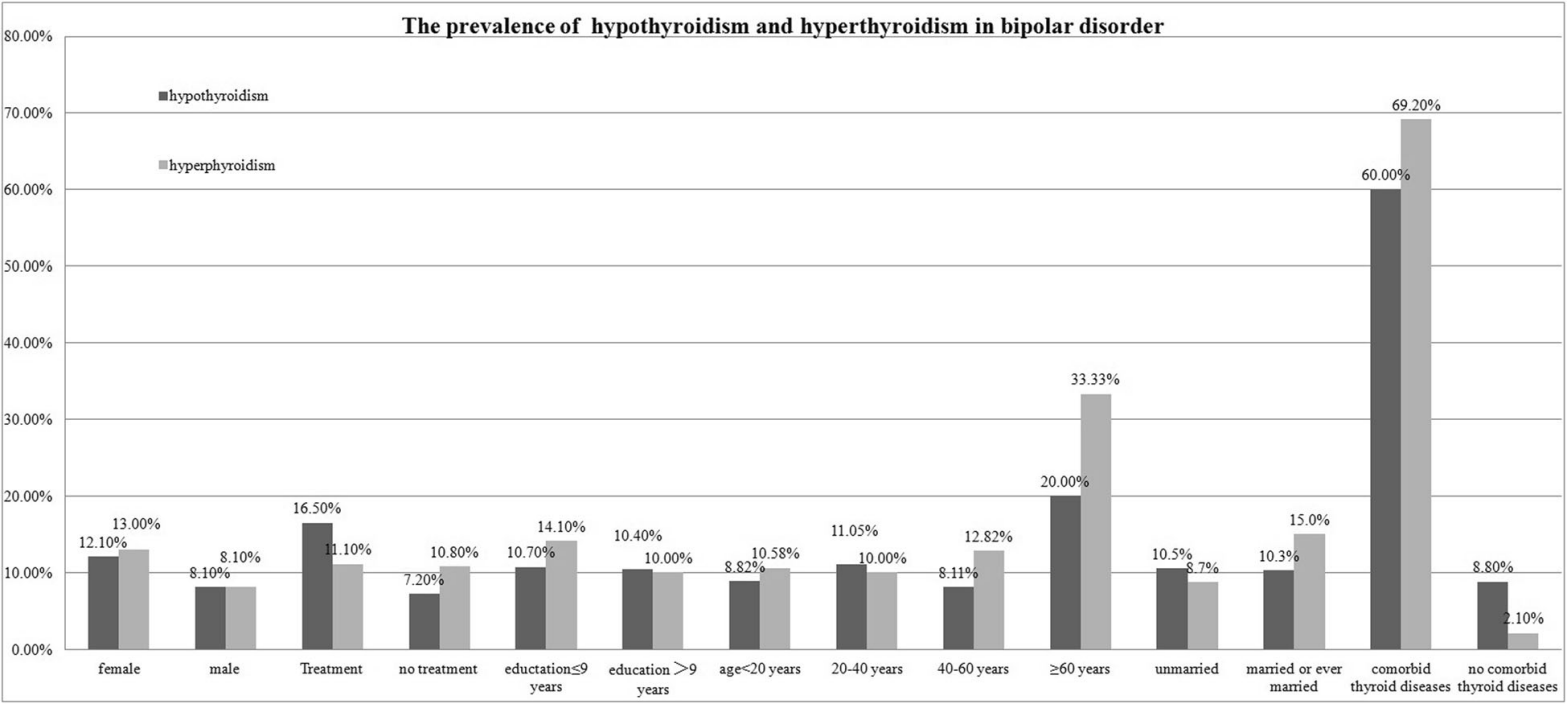
The prevalence of hypothyroidism and hyperthyroidism in bipolar disorder

In order to examine the association between hypothyroidism or hyperthyroidism and RC, patients with comorbidity of thyroid diseases were excluded. Totally, 333 eligible patients were included in the analysis, including 280 (84.1%) with normal thyroid function, 27 (8.1%) with hypothyroidism and 26 (7.8%) with hyperthyroidism. As seen in Fig. 1, the prevalence of hypothyroidism was higher among patients under psychopharmaceutical treatment than those without psychopharmaceutical treatment (*p* = 0.012), and both the prevalence of hypothyroidism and that of hyperthyroidism were higher among patients with comorbitidy of thyroid diseases than those without comorbidity of thyroid diseases. Although there was a tread for higher prevalence of hypothyroidism and hyperthyroidism among female subjects than among male ones, the difference did not reach significance (*p* > 0.10), even after adjusting for psychopharmaceutical treatment and comorbidity of thyroid diseases. In addition, married or ever married subjects also showed a higher prevalence of hyperthyroidism than those never married (*p* = 0.064), and the difference reached slight significance (*p* = 0.046) after controlling for psychopharmaceutical treatment and comorbidity of thyroid diseases. Education, marriage status and age did not significantly affect the prevalence of hypothyroidism or hyperthyroidism (*p* > 0.10).

#### The association between hypothyroidism or hyperthyroidism between RCBD

Univariable binary logistic regression was performed with rapid cycling (RC = 1, NRC = 0) as dependent variable and hypothyroidism (hypothyroidism = 1, normal thyroid function = 0) as independent variable. No significant association was found between RC and hypothyroidism (*p* = 0.481, OR = 0.452,95%CI = 0.123–2.387. After adjusting for gender, psychopharmaceutical treatment, the association still did not reach significance (*p* = 0.428). Similar statistical analysis did not find significant association between RC and hyperthyroidism (*p* = 0.847, OR = 0.884, 95%CI = 0.253–3.096) either and even after adjusting for gender, psychopharmaceutical treatment (*p* = 0.783).

### The association between TPO-abs or Tg-abs and RCBD

#### The prevalence of TPO-abs and Tg-abs positivity

**Fig. 2:**
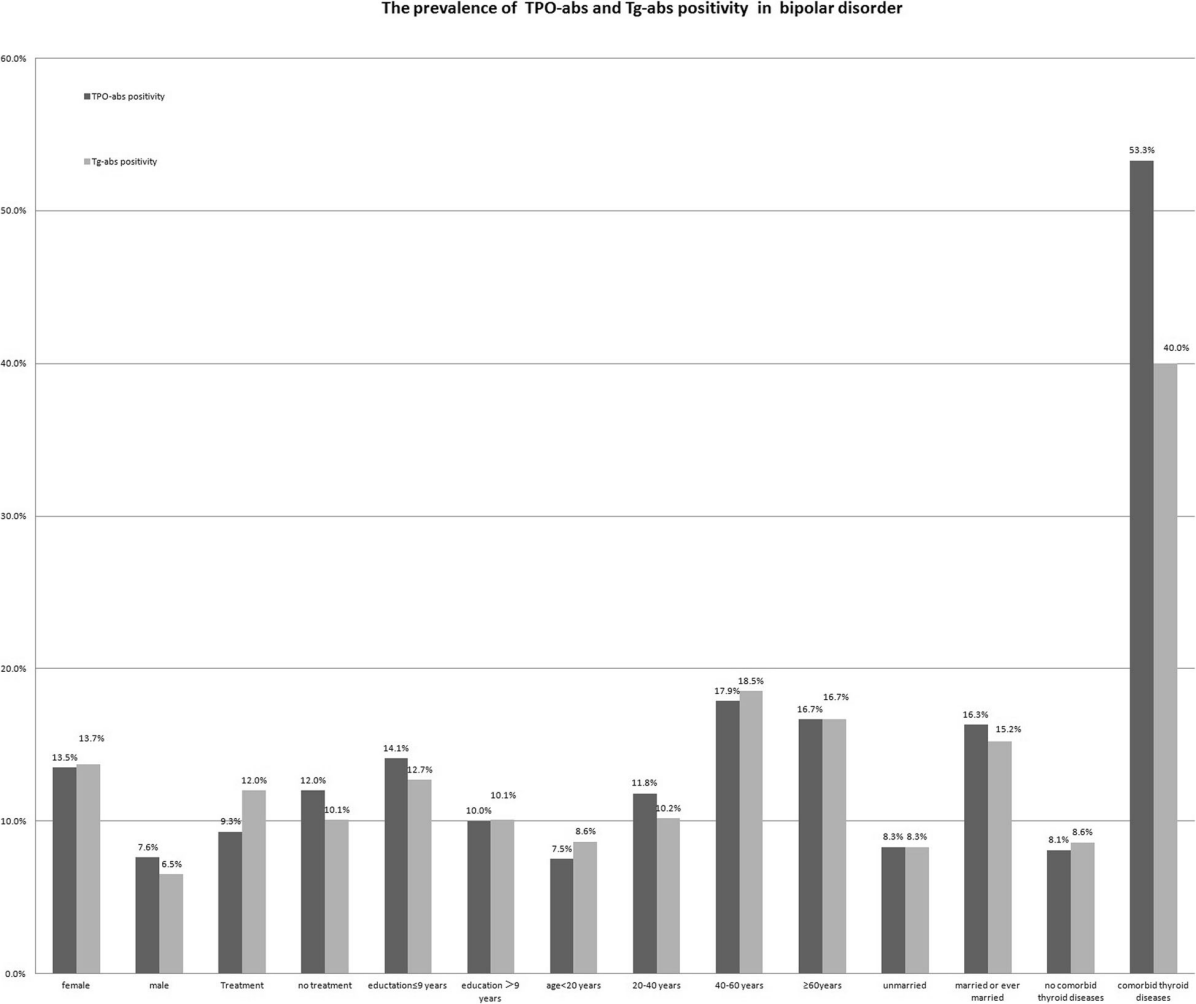
The prevalence of TPO-abs and Tg-abs positivity in bipolar disorder

Totally, 223 patients with the results of TPO-abs and Tg-abs were available to be analyzed here. The prevalence of TPO-abs positivity and Tg-abs positivity were 11.2% (25/223) and 10.8% (24/223) respectively. Figure 2 showed a significant higher prevalence of TPO-abs and Tg-abs positivity among patients with comorbidity of thyroid diseases than those without such comorbidity (*p* < 0.001). In addition, a trend for higher prevalence of TPO-abs and Tg-abs positivity was seen among female, less educated, elder, and married or ever married subjects, but all the differences did not reach significance (*p* > 0.10).

#### The relationship between TPO-abs and Tg-abs

Spearman’s correlation analysis found TPO-abs was significantly related to Tg-abs (*r* = 0.360, *p* < 0.001). Further univariate binary logistic regression with Tg-abs (1 = positivity, 0 = negativity) as dependent variable and TPO-abs (1 = positivity, 0 = negativity) as independent variable showed that Tg-abs positivity was significantly associated with TPO-abs positivity (*p* < 0.001, OR = 35.000, 95%CI = 12.089–101.332).

#### The association between antithyroid antibodies and RCBD

Univariate binary logistic regression was performed with rapid cycling (RC = 1, NRC = 0) as dependent variable and TPO-abs (positivity = 1, negativity = 0) as independent variable. The result showed that RC was significantly associated with TPO-abs (*p* < 0.001, OR = 5.130, 95%CI = 2.064–12.752), and the association remained significant after adjusting for gender, age, marriage status, education, antidepressants treatment, comorbidity of thyroid diseases, and thyroid function (serum levels of FT3, FT4 and TSH) (*p* = 0.003). Similar statistic analysis also found a significant association between RC and Tg-abs (*p* < 0.001, OR = 5.466, 95%CI = 2.177–13.723), and after adjusting for gender, age, marriage status, education, antidepressants treatment, comorbidity of thyroid diseases, and thyroid function (serum levels of FT3, FT4 and TSH), the association remained significant (*p* = 0.002).

Considering TPO-abs and Tg-abs were highly correlated with each other and they were both associated with RCBD as the above-mentioned results showed, multivariate binary logistic regression was conducted with rapid cycling (RC = 1, NRC = 0) as dependent variable, TPO-abs (positivity = 1, negativity = 0) and Tg-abs (positivity = 1, negativity = 0) as independent variables. Forward LR was used to select one of the independent variables that could represent the association with RC. It came out that TPO-abs was selected to maintain in the equation (*p* < 0.001, OR = 5.435, 95%CI = 2.165–13.646).

### The association between antithyroid antibodies and thyroid dysfunction

Univariate binary logistic regression was performed with hypothyroidism or hyperthyroidism as dependent variable, TPO-abs or Tg-abs as independent variables and psychopharmaceutical treatment as covariant to explore the relationship between antithyroid antibodies and thyroid dysfunction. TPO-abs positivity was found to be significantly associated with hyperthyroidism (*p* < 0.001, OR = 8.872, 95%CI = 3.454–22.788) but not with hypothyroidism (*p* > 0.10). Tg-abs was found to be marginally significantly related to hypothyroidism (*p* = 0.077, OR = 3.068, 95%CI = 0.886–10.619) but not with hyperthyroidism (*p* > 0.10). However, if adjusting for TPO-abs, the weak association between Tg-abs and hypothyroidism was not significant any more (*p* > 0.10), while if adjusting for Tg-abs, the association between TPO-abs and hyperthyroidism was still significant (*p* < 0.001, OR = 18.481, 95%CI = 4.510–75.739).

## Discussion

### The association between thyroid dysfunction and RC

In this study, we did not find a significant association between hypothyroidism or hyperthyroidism and rapid cycling, which was contrary to some previous reports [15, 16, 36–38], but consistent with a number of other studies [10, 23, 24, 39–42]. Instability of thyroid function might contribute to this inconsistence. Just as this study and other studies suggest, both comorbidity of thyroid diseases and antithyroid antibodies positivity [10] can affect thyroid function. Psychopharmaceutical treatment is another influencing factor. Beside lithium [43–45], many other medications such as quetiapine, risperidone, and aripiprazole, are reported to lead to hypothyroidism [46]. In addition, this study found a trend for higher prevalence of hypothyroidism in female and elder subjects, which was consistent with previous reports [47–49]. However, in the studies reporting a positive association between hypothyroidism and RCBD, most of the subjects were female, treated with lithium, had a history of thyroid diseases or consisted of a small sample [17, 50]. These limitations might be the reasons why their conclusion could not be replicated by later large scale studies.

### The association between antithyroid antibodies and RC

In this study, we found that both TPO-abs and Tg-abs were positively associated with RC, which was in line with Oomen’s report [22] that TPO-abs positivity (in particular with high titres and or with TSH > 4.0 mU/l) was significantly associated with RCBD. Thus, we provided further evident for a large national study’s finding that presence of thyroid disorder was one of the risk factors of developing RCBD [19]. One possible mechanism that may be involved in the association between TPO-abs and RCBD is the episodic alternating of hyperthyroidism (due to leakage of thyroid hormone from autoimmune-destructed thyrocytes) and hypothyroidism (due to a transient loss of functional thyrocyte mass) [22], since a neuromodulatory link has been found between the hypothalamic-pituitary-thyroid axis (HPT-axis) and serotonin [51–53] or β-adrenergic receptors [54] in the limbic region, a major part in charge of mood regulation. However, this study and other studies [26, 55] found that the association between TPO-abs with mental wellbeing was independent of thyroid function, suggesting that it seemed to be unlikely to act via this mechanism. Another possible mechanism for this association is that increased cytokines, produced in the immune reaction [56], act on the hypothalamic-pituitary-adrenal axis via receptors in the brain [57]. However, not all studies reported increased cytokines in RCBD [58, 59]. Therefore, to what extend this mechanism participates in this association remains unknown. A third possibility is that TPO-abs directly act on the corresponding antigen in the mood regulation center, since increased antithyroid antibodies levels have been detected in cerebrospinal fluid of patients with Hashimoto’s thyroiditis-associated encephalopathies [60] and TPO-abs can specifically bind to human astrocytes [61]. Furthermore, previous study suggests that antithyroid antibodies might directly affect brain function [62], though the exact mechanism is still unclear. The fact that the impact of antithyroid antibodies on mental wellbeing is independent of thyroid function, in return, adds to evident that antithyroid antibodies might act directly on the brain to affect the mood. Finally, vasculitis resulting in abnormalities in cortical perfusion might be another possible mechanism, since abnormalities in cortical perfusion were frequently found in a serial of BD patients with TPO-ab positivity [63]. Although not all the studies confirm the association between antithyroid antibodies and RC [10, 23], in these two studies, one [23] comprised a small sample (only 22 subjects), another’s prevalence of RC reached 42.9% (51.8% in female and 33.9% in male) [10], much higher than the 13.1% (46/352) found in this research and the 9% reported in a French national study [19], suggesting the representativeness of their sample was low.

### The association between TPO-abs, Tg-abs and thyroid dysfunction

In this study, we found that TPO-abs and Tg-abs were highly correlated with each other and both associated with RCBD. In the past, probably because Tg-abs was less specific for lymphocytic thyroiditis, TPO-abs was considered better marker for lymphocytic thyroiditis than Tg-abs. Therefore, TPO-abs was preferentially or solely used [10]. Although in this research, binary logistic regression with forward LR selected TPO-abs to remain in the equation, we still cannot tell which one really matters. However, from the perspective of the relationship between antithyroid antibodies and thyroid dysfunction, TPO-abs is more influencing than Tg-abs, since TPO-abs is significantly associated with hyperthyroidism and the association is independent of Tg-abs, but Tg-abs is only marginally significantly associated with hypothyroidism and such association must depend on the presence of TPO-abs, which was consistent with the previous view [64] that Tg-abs alone in the absence of TPO-abs were not usually associated with thyroid dysfunction.

### Strengths and limitations

Our study has the following strengths: First, it comprises one of the largest sample of BD patients ever examined. Second, thyroid function, TPO-abs and Tg-abs were measured simultaneously, which enable us to systematically examine their relationship. Third, most of ever-known potential confounding factors were excluded or adjusted in the process of statistics. Finally, the BD sample was well-defined by collecting as many clinical features as possible through face-to-face semi-structural interview. However, in interpreting our results it is important to realized that most of the thyroid function tests in our study were arranged on the next morning after admission to hospital, it therefore is possible that the prevalence of hypothyroidism be underestimated and that of hyperthyroidism be overestimated since transient mild elevations of free and total T4 have been commonly noted in acutely admitted psychiatric patients as a result of non-specific response to the stress of hospitalization [65, 66]. But, as noted above, it cannot explain the full picture of our results, such as TPO-abs being related positively to hyperthyroidism, and so seems an implausible explanation for all of our findings. But we cannot deny such possibility. Secondly, the cross-sectional design of the study precludes inferences about the causal relation between antithyroid antibodies or thyroid dysfunction and BD. Finally, our sample is relatively small, and will limit our power to detect any weak but potential important association.

## Conclusion

In sum, in a large well-defined BD population, we find that both TPO-abs and Tg-abs, instead of hypothyroidism or hyperthyroidism are significantly associated with RC, and such association is independent of thyroid dysfunction. TPO-abs is, independently of Tg-abs, related to hyperthyroidism while Tg-abs is marginally associated with hypothyroidism at the presence of TPO-abs. Although TPO-abs and Tg-abs are highly correlated with each other, TPO-abs seems to be more influencing in the association with RCBD and thyroid dysfunction. Therefore, TPO-abs might be treated as a biomarker of RCBD. Further exploring the underlying mechanism might help understand the nature of RCBD and find out new treatment target for it.

## Data Availability

The data that support the findings of this study are available on request from the corresponding author (Qi Zhang, Biotherapy Center, the Third Affiliated Hospital of Sun Yat-sen University, NO.600, Tianhe Road, Tianhe District, Guangzhou, China, 510630. Qizhang85253333@163.com). The data are not publicly available due to privacy or ethical restrictions.
